# A series of images of digestive cancers using Pill Cam COLON2 video capsule endoscopy

**Published:** 2014

**Authors:** C Busegeanu, A Filimon, A Stemate, L Negreanu

**Affiliations:** * Internal Medicine 2 Gastroenterology Department, University Hospital; “Carol Davila” University of Medicine and Pharmacy, Bucharest

**Keywords:** colon cancer, colonoscopy failure, colonoscopy refuse, colon capsule endoscopy, PillCam Colon 2

## Abstract

Colon capsule endoscopy is regarded as an option to complement or even replace diagnostic colonoscopy in selected cases.

Since capsule lacks the capability of taking biopsies, a diagnosis of colon cancer usually requires a further confirmation by colonoscopy.

A series of seven patients who had highly suspicious lesions at capsule endoscopy (five-colon tumors, one gastric tumor and one small bowel tumor) and in whom the clinical decision and treatment was solely based on capsule findings, are presented. The diagnosis of cancer was confirmed in all cases by surgery and histology.

Conclusion: In selected patients with a high index of clinical suspicion of cancer, PillCam colon 2 capsule endoscopy might be a sufficient tool for diagnosis.

## Introduction

Colon capsule endoscopy (CCE) with PillCam Colon was developed especially to increase the acceptability and safety of colonic examination.

With the advent of the second-generation, improved, CCE system (PillCam Colon 2), the sensitivity for colorectal polyp detection significantly increased compared with the first-generation system [**1**].

Several studies including the ones from our center, showed the effectiveness of colon capsule in diagnosing significant lesions (e.g. polyp ≥ 6 mm), in patients in whom colonoscopy was either contraindicated or refused [**2**].

**Aim**

The capsule images from seven cases with digestive cancer were presented. The importance of capsule examination on the clinical decision and follow up was emphasized.

**Procedure**

The second generation Pill Cam Colon 2 capsule and Rapid 7 reading software were used in the study.

Colonic preparation and boosters were realized by using Macrogol 4000 (Fortrans®- Beaufour Ibsen, France).

In order to obtain images of the entire digestive tract, capsule was set up to record starting from the moment of ingestion.

**Case series**

All the patients underwent capsule after a previous failure of a colonoscopy or if they refused a colonoscopy examination.

Five patients had colon tumors detected by CCE.

A 62-year-old woman with colon cancer family history, presented anemia due to iron deficiency and failure of colonoscopy, in another center.

**Fig. 1:**
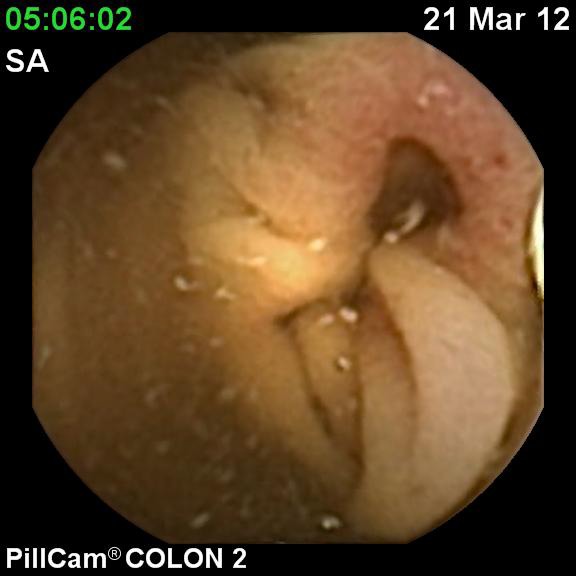
Ulcerated stenosis in the descedending colon

The CCE revealed a left flexure stenotic tumor with capsule failing to pass the stenosis (**Fig. 1**).

Another colonoscopy with confirmation biopsies was discussed with the patient but she preferred to undergo surgery directly. A left colectomy was performed uneventfully. The patient underwent six cycles of chemotherapy. After one year, she had a normal colonoscopy and CT scan.

A 38-year-old man with a family history of colon cancer and iron deficiency anemia was referred from another department for small bowel capsule, where upper and lower endoscopies were stated as normal.

**Fig. 2:**
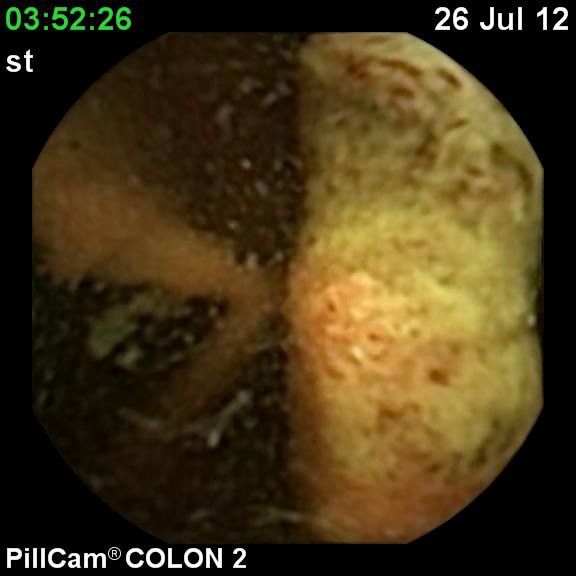
Ulcerated tumor

We agreed with the patient to use Pillcam Colon 2, which discovered two synchronous lesions in the cecum and ascending colon (**Fig. 2**), one with significant stenosis and capsule impaction above the lesion until battery depletion. Another colonoscopy under general sedation confirmed the presence of the lesions located at the hepatic flexure and cecum. Since we could not review his previous colonoscopy, we could only suppose that it was mistakenly interpreted as complete and normal due to poor colonic preparation. The patient had surgery and started chemotherapy and he is currently seen in our center.

**Fig. 3:**
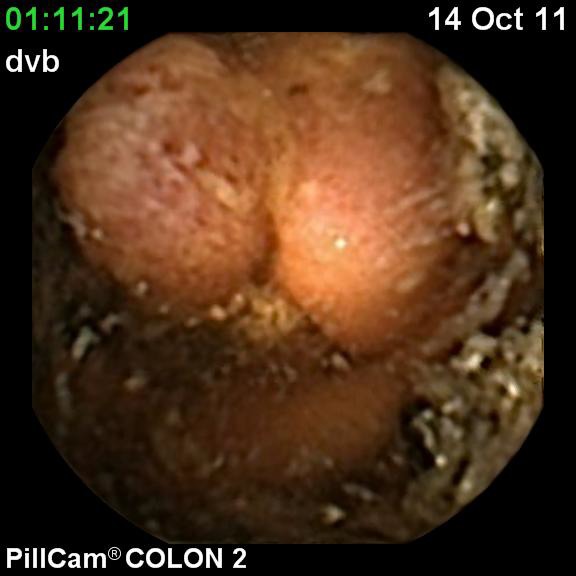
Ulcerated cecal tumor

A 66-year-old overweight female was admitted for iron deficiency anemia and chronic diarrhea. She refused to undergo colonoscopy. An ulcerated cecal tumor was discovered by capsule examination (**Fig. 3**). After CT scan, surgery was performed and diagnosis adenocarcinoma was confirmed. She started chemotherapy and she is now free of disease at two years.

**Fig. 4:**
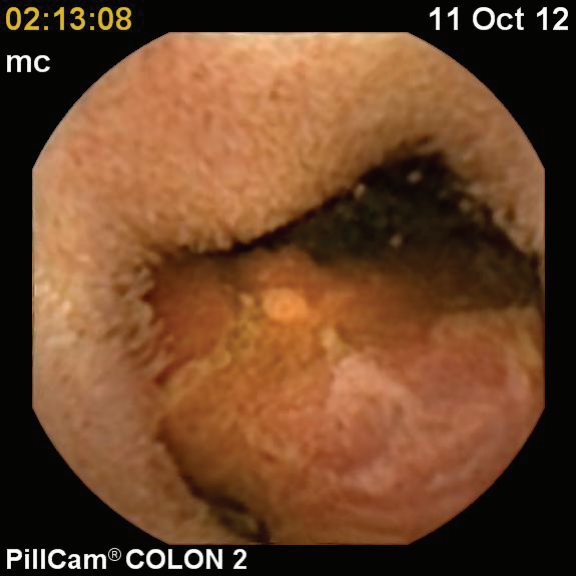
Ulcerated cecal tumor: transvalvular image from small bowel side

A 65-year-old female was admitted for iron deficiency anemia. After normal upper endoscopy and failure of colonoscopy due to diverticula in the sigmoid colon, the CCE showed a tumor located in the cecum (**Fig. 4**). Surgery was successfully performed and confirmed an ulcerated adenocarcinoma of the cecum. The patient was lost from follow up 9 months after surgery.

**Fig. 5:**
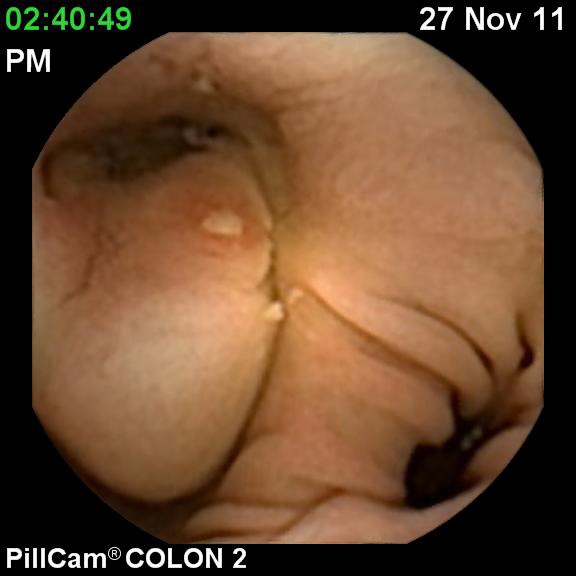
Submucosal mass with surface mucosal ulcerations

A 75-year-old male patient with left lower quadrant abdominal pain, weight loss and chronic diarrhea was referred for a second opinion. He had been admitted in a different department in the previous month, where he underwent an upper endoscopy that found no lesions and two colonoscopies attempts, which failed to pass the sigmoid colon. After a barium enema, he was diagnosed with diverticulosis and its symptoms interpreted as a diverticulitis attack. Since he had two colonoscopy failures before, we decided to use CCE for the colonic exploration. A complete colonic examination showed multiple diverticula with no signs of inflammation of the surrounding mucosa and an ulcerated submucosal mass of approximately 2 cm in diameter located in the sigmoid (**Fig. 5**).

A contrast CT scan showed multiple colonic diverticula with minimal inflammation signs and a soft tissue mass of 5cm diameter with a necrotic centre, with a surrounding thickened sigmoid wall and densification of the perisigmoid fat and with no clear demarcation with the urinary bladder. This kind of necrosis is quite specific and previously described in GIST’s [**3**].

Surgery with immunochemistry confirmed the diagnosis of locally invading GIST. Imatinib mesylate therapy was started and patient was free of disease at 12 months.

Two other digestive tumors were discovered by the CCE examination.

**Fig. 6:**
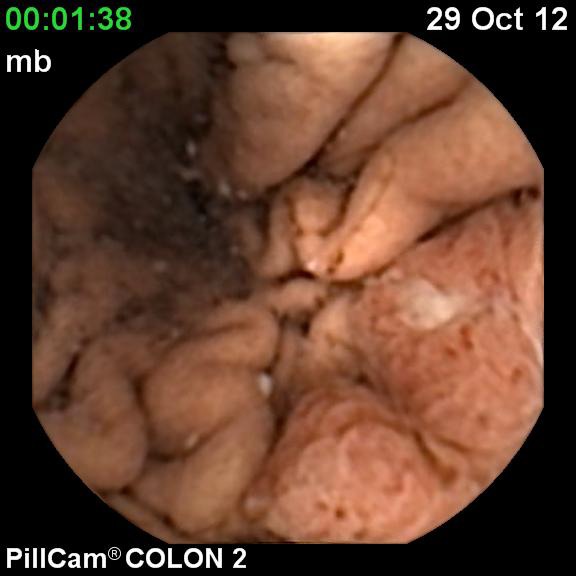
Ulcerated gastric tumor

In a 58-year-old male patient with iron deficiency anemia, suspect CT scan (abdominal mass in the left upper quadrant in close contact both stomach and colonic walls) and failure of colonoscopy, an ulcerated lesion was discovered by the capsule in the stomach. An upper endoscopy with biopsies established the diagnosis of undifferentiated gastric cancer (**Fig. 6**). He had surgery with successful gastrectomy and started chemotherapy but died due to pulmonary complications seven months later.

**Fig. 7:**
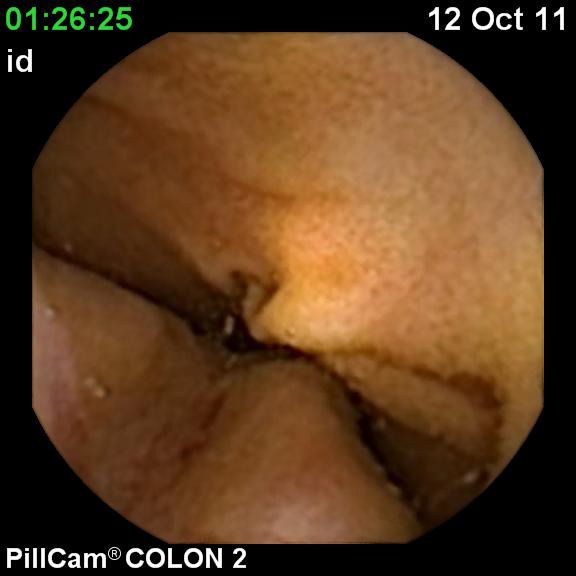
Voluminous jejunal tumor

A 75-year-old woman also referred for anemia and suspect imaging (mass seen on ultrasound). She had a normal upper endoscopy and refused colonoscopy but accepted to undergo colon capsule examination. An ulcerated tumor of the small bowel was visualized in the jejunum (**Fig. 7**). She had surgery, which confirmed the diagnosis of GIST. She is currently under supervision in another center.

## Discussion

According to the European Society of Gastrointestinal Endoscopy colon capsule endoscopy guidelines, CCE is feasible and safe and appears to be an accurate screening tool when used in average-risk individuals. A CCE based screening may be cost-effective if it will increase patient uptake when compared with colonoscopy [**4**].

Colonoscopy should be the first choice in high-risk patients (alarm symptoms or signs, family or personal history of CRC), who are at increased risk of advanced colorectal neoplasia or cancer.

However, in patients for whom colonoscopy is inappropriate or not possible, the use of CCE could be discussed with the patient [**4**].

We are aware that our experience is quite unusual. Several particularities should also be mentioned.

This is a case series of high-risk patients with a high index of suspicion (symptomatic, family history of CRC, other suspect imaging) in which capsule endoscopy was used as an alternative to a failed or refused colonoscopy.

The activation of recording starting from the capsule ingestion allowed the discovery of two other extra-colonic tumors. Also it showed that the current quality criteria in investigating such patients were not followed: gastric lesion in one, and colonic cancer in another in whom a previous “complete” colonoscopy was normal. We believe that in these patients, a throughout endoscopic examination should be realized together with documented-images and videos.

After a first colonoscopy failure, different alternatives might be proposed to the patient: different center, different colonoscopist, use of an enteroscope, CT colonography. In our center, after failure of a first colonoscopic examination, patients usually undergo another colonoscopy with better sedation by an experienced operator. Colon capsule endoscopy can be a valuable alternative in selected cases.

In the patients presented, the capsule results had an important role for the clinical decision and treatment. The results were confirmed after surgery and histology in all cases.

## Conclusion

Establishing a diagnosis of digestive cancer after capsule endoscopy examination is not current practice. It is not our intention to propose such an approach.

The PillCam Colon 2 was effective in detecting lesions with a high index of suspicion of cancer in symptomatic patients who either failed or refused a colonoscopy. The activation of the recording from the beginning allowed the detection of stomach and small bowel tumors.

A “capsule” approach to selected patients might be very useful.

**Conflicts of interests: None.**

**Author contributions:** authors equally contributed to the article.

**Acknowledgements:** This paper is partly supported by the Sectorial Operational Program Human Resources Development SOPHRD financed by the European social fund and the Romanian government under the contract number POSDRU 141531
